# Giant cell tumor of bone in the mandible presenting without typical histological features: a case report

**DOI:** 10.1186/s13256-025-05601-8

**Published:** 2025-10-21

**Authors:** Kaori Oya, Kouhei Kawamura, Ryou Akiyama, Hiroki Kiyokawa, Toshihiro Uchihashi, Hiroaki Shimamoto, Tadashi Sasai, Shin-Ichiro Hiraoka, Shumei Murakami, Satoru Toyosawa

**Affiliations:** 1 Department of Clinical Laboratory, The University of Osaka Dental Hospital, 1-8 Yamadaoka, Suita, Osaka 565-0871 Japan; 2 Department of Oral and Maxillofacial Surgery, Graduate School of Dentistry, The University of Osaka, 1-8 Yamadaoka, Suita, Osaka 565-0871 Japan; 3 Department of Pathology, Graduate School of Medicine, The University of Osaka, 1-8 Yamadaoka, Suita, Osaka 565-0871 Japan; 4 Department of Oral and Maxillofacial Radiology, Graduate School of Dentistry, The University of Osaka, 1-8 Yamadaoka, Suita, Osaka 565-0871 Japan; 5 Department of Oral and Maxillofacial Pathology, Graduate School of Dentistry, The University of Osaka, 1-8 Yamadaoka, Suita, Osaka 565-0871 Japan

**Keywords:** Giant cell tumor of bone, Mandible, H3.3G34W immunostaining, Case report

## Abstract

**Background:**

Giant cell tumor of bone is a locally aggressive bone tumor characterized by the proliferation of round-to-oval mononuclear cells and uniformly distributed osteoclast-type giant cells. Giant cell tumor of bone typically arises in long bones, whereas craniofacial involvement is rare. Atypical histological and clinical presentations can complicate diagnosis. This study presents a challenging case of giant cell tumor of bone in the mandible.

**Case presentation:**

A Japanese man in his 70s presented with a slowly expanding radiolucent lesion in the left mandible, first noted a decade ago, with no subjective symptoms. Cone-beam computed tomography revealed a 27 mm × 12 mm × 23 mm radiolucent lesion with irregular borders and discontinuity of the mandibular canal. Excisional biopsy showed the proliferation of bland spindle cells with small multinucleated cells, which indicated central giant cell granuloma. However, the spindle cells were positive for H3.3G34W, a specific marker of giant cell tumor of bone, which confirmed the diagnosis of giant cell tumor of bone. Conventional histological features of giant cell tumor of bone were absent throughout the observation period.

**Conclusion:**

Morphological analysis alone is insufficient for diagnosing giant cell tumor of bone, and H3.3G34W immunostaining is valuable in distinguishing it from other giant cell lesions. The possibility of giant cell tumor of bone should not be ruled out in cases involving the jaw, although its occurrence is rare.

## Background

Giant cell tumor of bone (GCTB) is a locally aggressive, intermediate malignancy of the bone, characterized by the proliferation of round-to-oval mononuclear cells and uniformly distributed osteoclast-type giant cells with numerous (50–100) nuclei [1, 2]. The neoplastic mononuclear cells of GCTB highly express the receptor activator of nuclear factor kappa B (RANK) ligand (RANKL), which binds to RANK expressed on mononuclear osteoclast precursors. The RANK–RANKL interaction results in giant cell formation and the osteolytic nature of the tumor [3]. GCTB represents 4–5% of all primary bone tumors, with a peak incidence in individuals of 20–45 years of age [1, 4]. The tumor typically arises at the ends of long bones in the mature skeleton, and craniofacial skeleton involvement is rare [1, 2, 5, 6].

Along with the classic GCTB morphology, atypical morphological patterns include fibrosis, bone or cartilage formation, cystic changes, necrosis, and secondary aneurysmal bone cyst (ABC) formation. Mitoses ranging from 0 to 35 per ten high-power fields have been observed [5, 7]. Additionally, the disease can occur in diverse age groups. Previous studies indicate that GCTB occurs in 8.9% of patients younger than 18 years and 4.8% of those older than 55 years [5, 7]. Moreover, case reports of craniofacial skeleton involvement exist, albeit with rarity or information deficiency [8–10]. Diagnostic challenges may arise when GCTB without typical and classical histological features develops at unusual sites or in uncommon age groups. Herein, we present a challenging case of GCTB involving the mandible.

## Case presentation

**Fig. 1:**
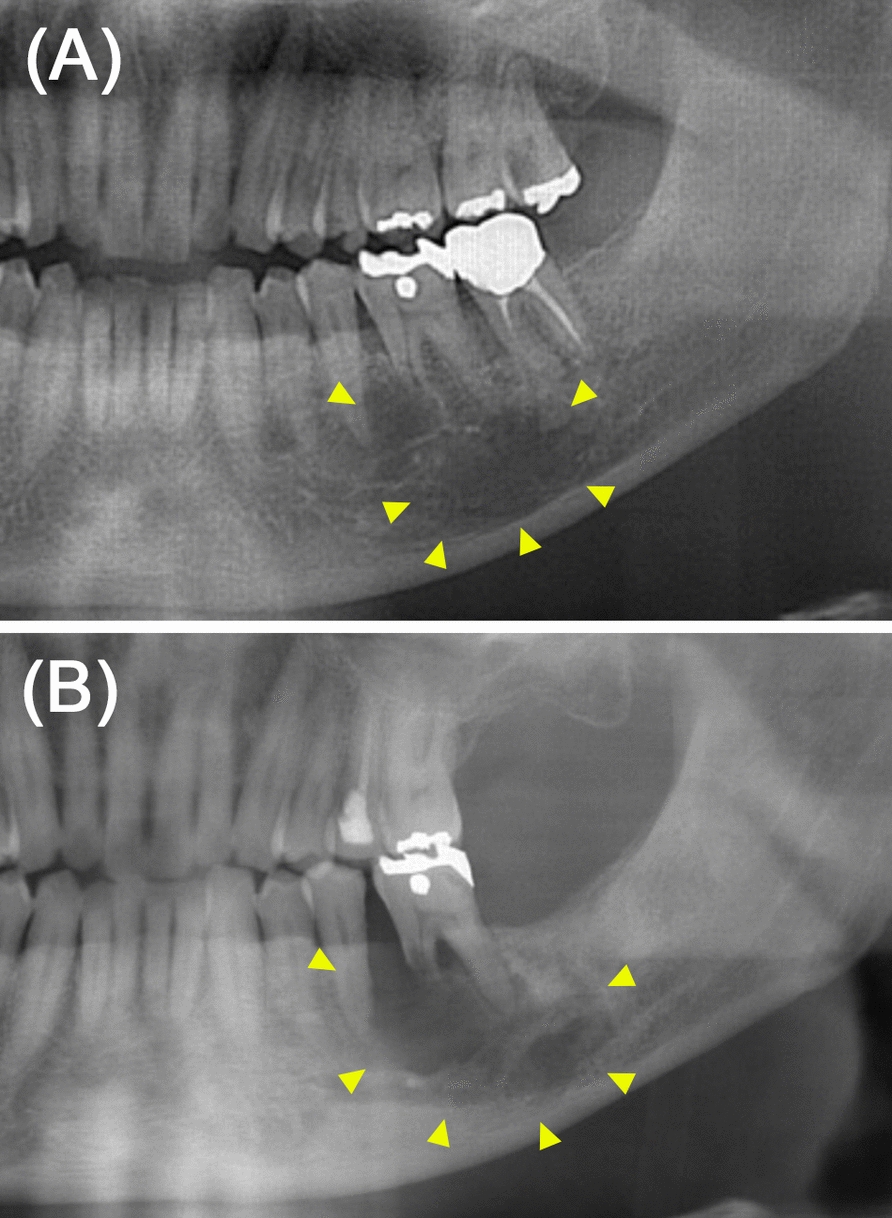
Comparison of panoramic radiographs. Panoramic radiographs taken **A** 10 years ago at the family dental clinic and **B** during the first visit to this hospital. The unclear radiolucent lesion around the root apex of the left first molar of the mandible (**A**, arrowhead) became clear with medial root absorption of the first molar (**B**, arrowhead)

**Fig. 2:**
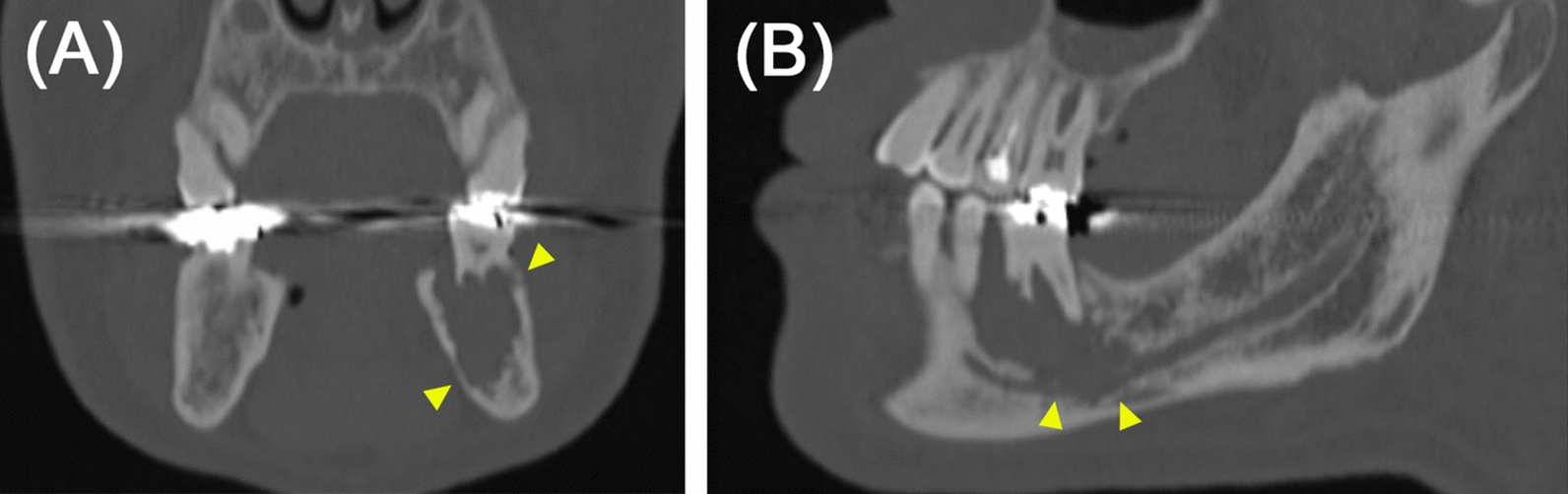
Imaging findings of the primary lesion. Coronal cone-beam computed tomography image shows a radiolucent lesion with an irregular border and no expansion of the cortical bone (**A**, arrowhead). Sagittal cone-beam computed tomography image shows the disappearance of the mandibular canal wall (**B**, arrowhead)

**Fig. 3:**
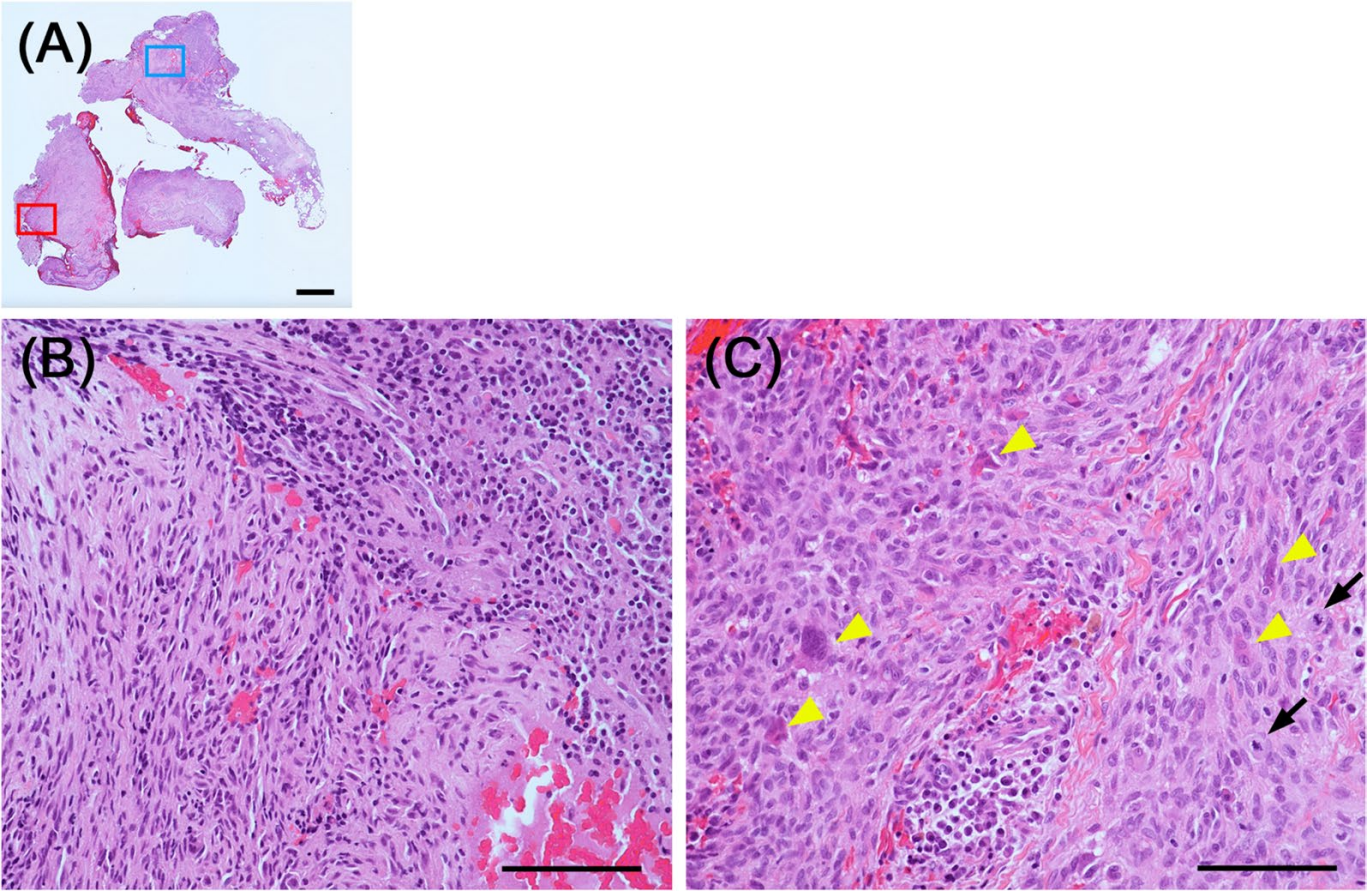
Histological findings of the biopsied specimen. **A** Low magnification image of the hematoxylin and eosin-stained specimen (2× magnification). **B** High magnification image of the blue box in (**A**). It consisted mainly of inflammatory granulation tissue with hemorrhage. **C** High magnification image of the red box in (**A**). The proliferation of spindle cells with small multinucleated cells (arrowhead) was observed. Cellular atypia was absent in the spindle cells. Two mitoses (arrow) were observed (20× magnification). Scale bar: (**A**) 1000 μm, (**B**) and (**C**) 100 μm

A Japanese man in his 70s presented to our hospital for a close examination of an expanding radiolucent lesion in the left mandible, first noted approximately a decade ago, with no symptoms (Fig. 1). His medical history included hypertension, gout, and hyperlipidemia, for which he was on medication. Cone-beam computed tomography (CBCT) revealed a radiolucent lesion measuring 27 mm × 12 mm × 23 mm with an irregular border and discontinuity of the mandibular canal (Fig. 2). His serum level of the squamous cell carcinoma antigen was elevated at 3.4 ng/mL (1.5 ng/mL). These findings suggest the possibility of a primary intraosseous carcinoma; however, a prolonged course and absence of symptoms were unsupportive for this diagnosis. Extraction of the affected first molar and excisional biopsy were performed. Hematoxylin and eosin (HE)-stained sections of the biopsied specimens showed that most of the tissue consisted of inflammatory granulation tissue with hemorrhage (Fig. 3A, B). However, the proliferation of the spindle cells with small multinucleated cells was also observed (Fig. 3C). Cellular atypia was absent in spindle cells, and a few mitoses were also observed (two per ten high-power fields). Central giant cell granuloma (CGCG) was the most likely diagnosis because of the histology and site of origin, although the possibility of reactive lesion was considered due to the high proportion of granulation tissue. The differential diagnosis also included ABC because of its histological similarities and hemorrhagic properties. Although GCTB was considered unlikely because its occurrence in the craniofacial skeleton is quite rare, it was listed to be ruled out because the histological findings of the present case were similar to one of the histological variants of GCTB.

**Fig. 4:**
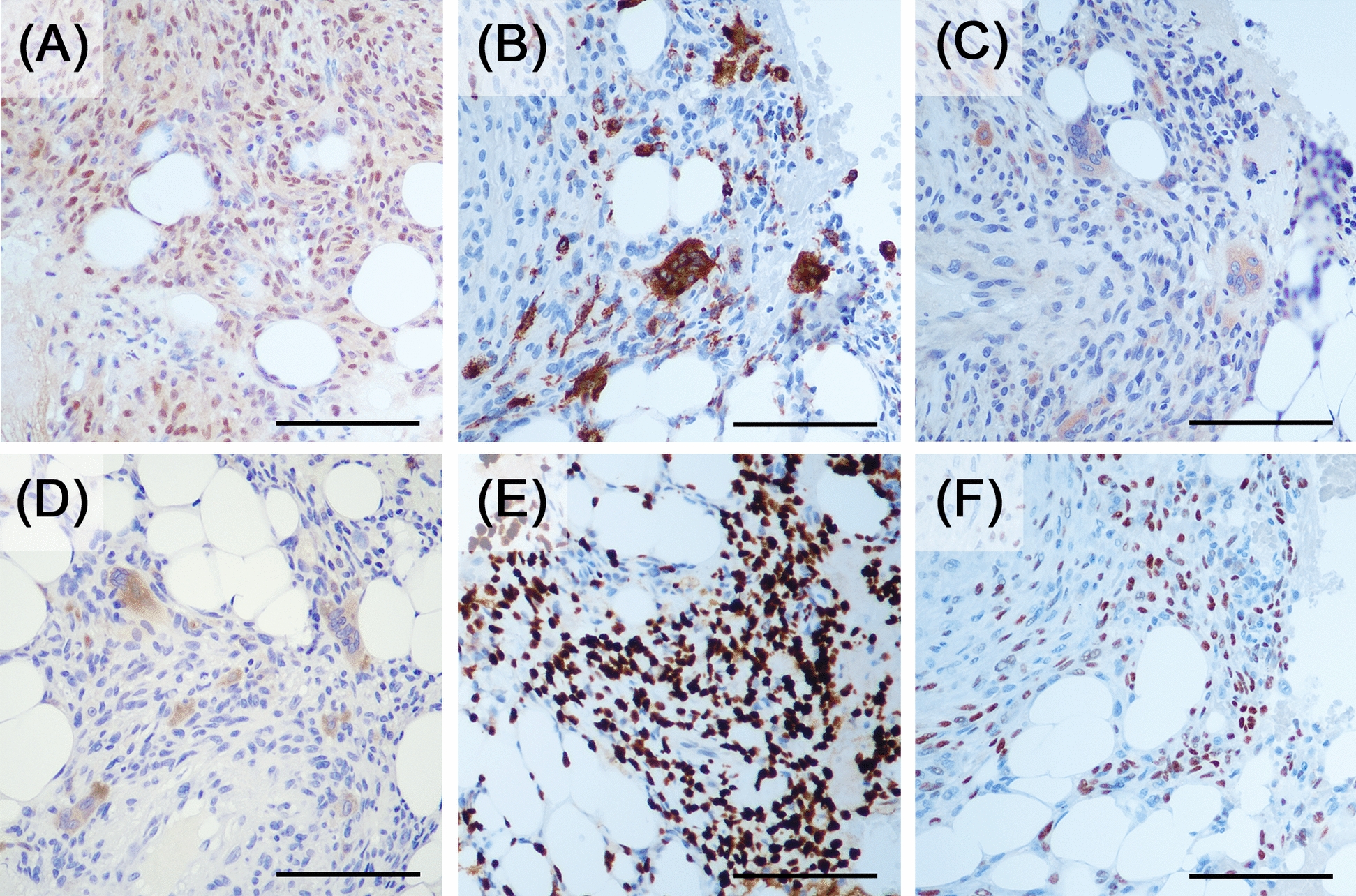
Immunohistochemical staining results. (**A**) RUNX2, (**B**) CD68, (**C**) TRAP, (**D**) cathepsin K, (**E**) H3.3G34W, and (**F**) p63 (20× magnification). Scale bar: (**A**–**F**) 100 μm

Spindle cells were positive for runt-related transcription factor 2 (RUNX2), a marker of osteogenesis. Low proliferative activity was confirmed using Ki-67 immunostaining (< 3%). The small multinucleated cells were positive for cluster of differentiation 68 (CD68), tartrate-resistant acid phosphatase (TRAP), and cathepsin K, which are markers of osteoclasts. These results were consistent with the characteristics of CGCG. However, the spindle cells were also positive for H3.3G34W and p63, which are markers of GCTB (Fig. 4). Therefore, a final diagnosis of GCTB was established

**Fig. 5:**
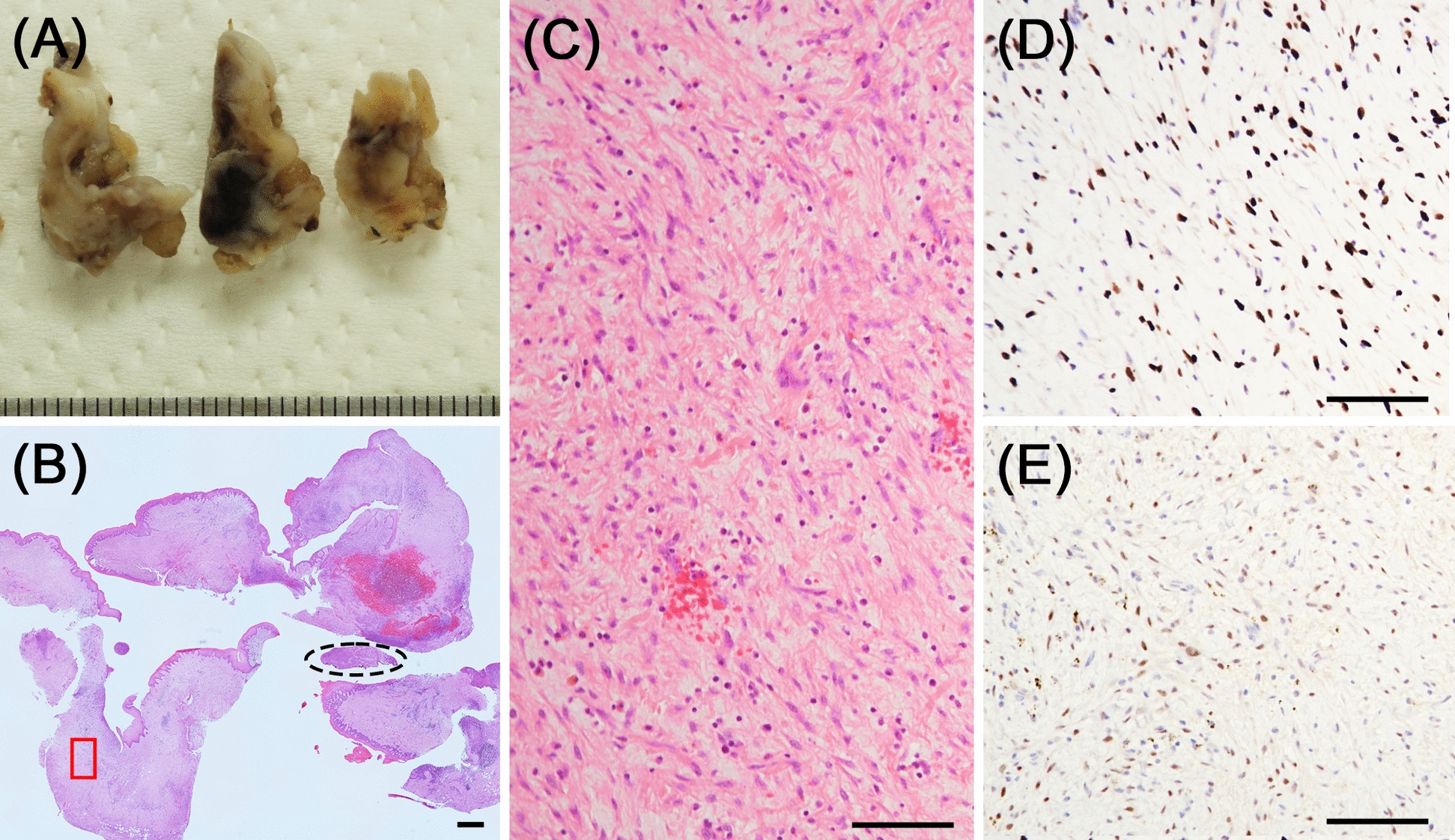
Gross appearance and histological findings of the resected specimen. **A** Macroscopically, the lesion appeared as a white soft mass with partial bleeding. **B** Low magnification image of the hematoxylin and eosin-stained specimen. Spindle cell proliferation with high cell density was observed only in a very small area (dotted line frame) (2× magnification). **C** High magnification image of the red box in (**B**). The main component showed a granulation tissue-like appearance (10× magnification). **D** and **E** Immunohistochemical staining results for (**D**) H3.3G34W and (**E**) p63 corresponding to (**C**). Fibroblast-like spindle cells in the granulation tissue-like area were positive for them (10× magnification). Scale bar: (**A**) 1000 μm, (**C**) and (**D**) 100 μm

Under general anesthesia, curettage was performed, and the specimen was subjected to histopathological examination. After formalin fixation, the lesion appeared as a soft white mass with partial bleeding (Fig. 5A). Histological examination with HE staining revealed that the main component had a granulation tissue-like appearance (Fig. 5B, C). Spindle cell proliferation with high cellular density was observed in only a very small area (Fig. 5B). At first glance, it appeared that the lesion had not been scraped out; however, immunostaining for H3.3G34W and p63 revealed that most of the tissue consisted of tumor cells. Many fibroblast-like spindle cells in the granulation tissue-like area were positive for them (Fig. 5C–E). Typical histological features of GCTB were not observed.

Follow-up CBCT examination at 18 months post-surgery showed no evidence of recurrence, and the patient showed satisfactory progress.

## Discussion and conclusion

The recent finding of H3.3 histone A (*H3F3A*) gene mutations in 92% of GCTB cases [11] has improved GCTB diagnostic ease and certainty, with the detection of the mutated proteins H3.3G34W, G34R, and G34V using immunostaining [3]. Particularly, the usefulness of immunostaining for H3.3G34W, the most frequent variant, has been reported. A specificity and positive predictive value of 100% has been demonstrated [12], with sensitivities ranging from 85% to 90.6% [13, 14].

**Fig. 6:**
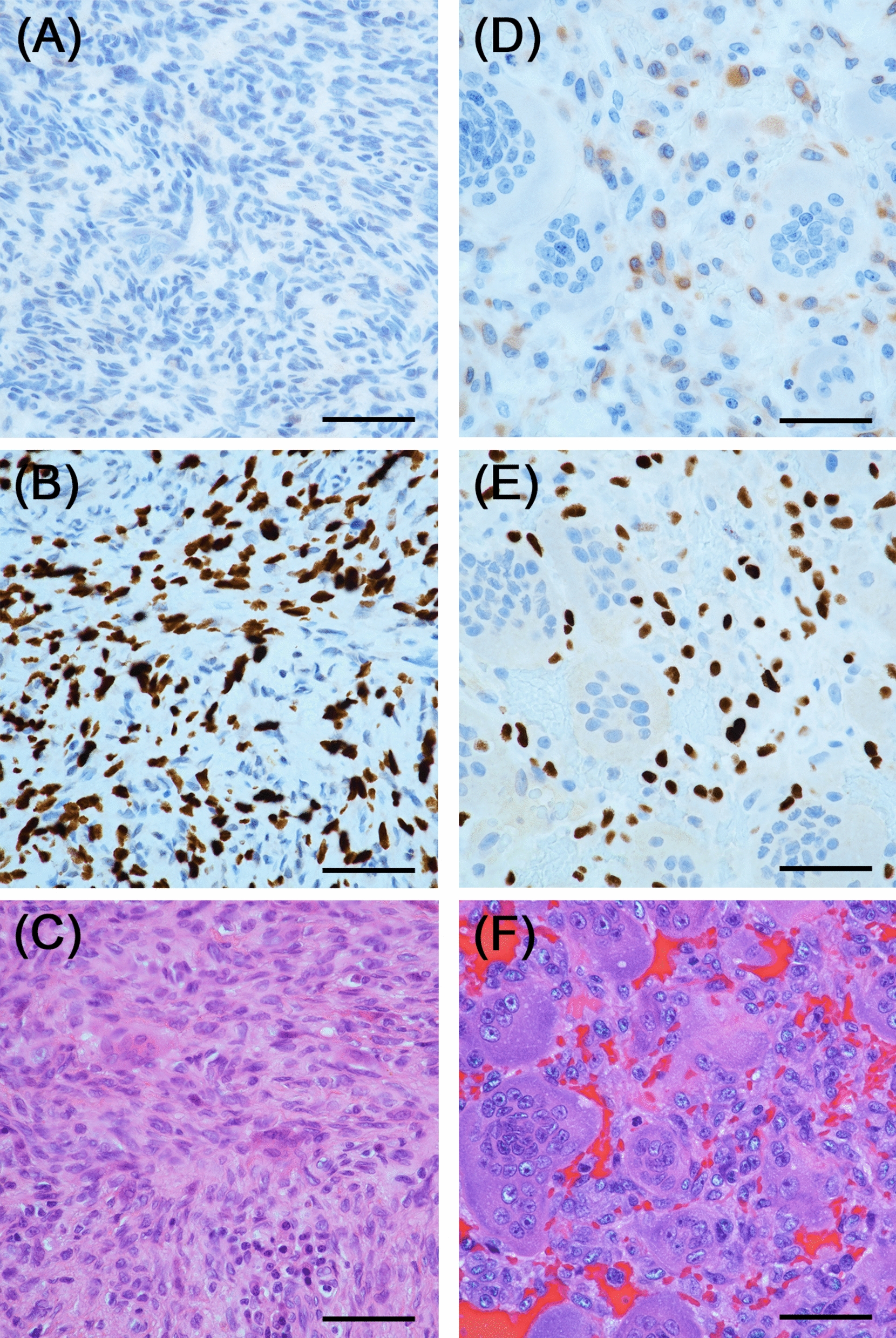
Comparison of the present case (**A–C**) and typical giant cell tumor of bone case (**D–F**). **A** Receptor activator of nuclear factor kappa B ligand expression scarcely be observed in the present case, but (**D**) mononuclear cells in typical giant cell tumor of bone case clearly express receptor activator of nuclear factor kappa B ligand. **B** and **E** Immunostaining results for H3.3G34W and (**C**) and (**F**) hematoxylin and eosin-stained images corresponding to (**A**) and (**D**), respectively. (40× magnification). Scale bar: (**A–D**) 50 μm

GCTB can typically be diagnosed through histological examination of the entire lesion site, as most GCTB cases exhibit conventional morphology, although several histological variants have been reported [5, 6]. A previous report of a GCTB lesion predominantly composed of spindle cells with no giant cell involvement was introduced in the Armed Forces Institute of Pathology atlases, although the patient was initially misdiagnosed as having benign fibrous histiocytoma [2]. Herein, we present a case of GCTB without typical histological features. In ours as well as in the previously reported case, immunohistochemical staining for H3.3G34W proved crucial for diagnosing GCTB. Similar to ours, other cases may be misdiagnosed due to the lack of typical features and examination for *H3F3A* gene mutations [2]. Therefore, H3.3G34W immunostaining should be performed even if the diagnosis is considered to be CGCG and others showing spindle cell proliferation. Although the reason why giant cells are less obvious in these cases remains undetermined, a reduction of RANKL expression is one possible reason. The presented case showed a significant reduction in RANKL positivity compared with typical GCTB cases in immunostaining (Fig. 6). Identifying similarities among such cases can help elucidate the mechanism.

*H3F3A* mutations or H3.3G34W immunopositivity have not been observed in giant cell lesions and bone cysts of the jaw [12, 14, 15], which supports the rarity of GCTB in the jawbone. However, our case report involved the incidence of GCTB in the mandible. The possibility of GCTB should not be ruled out on the basis of its site of occurrence, as GCTB can occur in any bone [3].

The most common differential diagnoses of giant cell lesion include CGCG, ABC, and non-ossifying fibroma (NOF), in addition to GCTB [1, 16]. Morphological similarities complicate diagnosis; however, immunohistochemical staining, genetic testing, and clinical information aid in achieving accuracy. Aside from H3.3G34W, other useful markers exist. p63 immunopositivity is observed in GCTB but not CGCG [17], although both share common features such as high RUNX2 expression [18]. As approximately 75% of ABC cases harbor a chromosomal translocation involving the *USP6* gene with various fusion partners [19, 20], fluorescence in situ hybridization testing for *USP6* rearrangements can be used for diagnosis. NOF exclusively arises in skeletally immature individuals, with a peak incidence in the second decade of life, and affects the metaphyses of long bones [1, 16]. Compatible imaging findings, including well-defined, lobulated radiolucent lesions centered in the cortical bone with sclerotic and scalloped borders, are essential for the diagnosis of NOF [1].

The standard treatment for GCTB is curettage [2], although recurrence has been observed in 15–50% of cases [1]. Wide resection is associated with a lower recurrence rate than intralesional surgery [21] but can cause significant morbidity [3]. Polymethylmethacrylate may reduce local recurrence risk [21]. Denosumab, an inhibitor of RANKL, may be considered for unresectable and advanced cases [1]. It suppresses bone resorption by inhibiting the formation of osteoclast-like giant cells by blocking the RANK–RANKL interaction. Hayashida *et al*. reported that local recurrence cannot be reduced by denosumab treatment [22]; however, no consensus regarding its use exists [1]. Denosumab changes the histomorphology of GCTB; complete elimination of osteoclast-like giant cells, fascicular and storiform patterns of spindle-shaped mononuclear cells, sometimes mixed with foam cells, and reticular woven bone formation, have been observed [23]. Thus, it is crucial to refer to clinical history in the diagnosis of such cases to prevent misidentification of other fibro-osseous lesions or spindle cell sarcoma [24]. In this case, only intralesional curettage was performed. As a risk of recurrence exists, we intend to follow up on the case closely.

In conclusion, we report a case of GCTB resembling CGCG of the mandible. Morphological analysis alone is insufficient for diagnosis; detecting *H3F3A* mutations or mutated proteins is essential. H3.3G34W immunostaining is valuable for distinguishing GCTB from other giant cell lesions in daily clinical practice. Although rare, GCTB should remain a differential consideration in jaw lesions.

## Data Availability

The datasets used and/or analyzed during the current study are available from the corresponding author on reasonable request.

## Abbreviations

GCTB: Giant cell tumor of bone
RANK: Receptor activator of nuclear factor kappa B
RANKL: Receptor activator of nuclear factor kappa B ligand
ABC: Aneurysmal bone cyst
CBCT: Cone-beam computed tomography
HE: Hematoxylin and eosin
CGCG: Central giant cell granuloma
RUNX2: Runt-related transcription factor 2
CD68: Cluster of differentiation 68
TRAP: Tartrate-resistant acid phosphatase

## References

[1] Bovee JVMG, Flanagan AM, Lazar AJ, Nielsen GP, Yoshida A. WHO classification of tumours, Soft tissue and bone tumours. 5th ed. Lyon: IARC; 2020.

[2] Nielsen GP, Rosenberg AE, Bovee JVMG, Bredella MA, Ferry JA. AFIP atlases of tumor and non-tumor pathology, Series 5 Tumors of the bones and joints. Arlington: ARP; 2021.

[3] Noh BJ, Park YK. Giant cell tumor of bone: updated molecular pathogenesis and tumor biology. Hum Pathol. 2018;81:1–8. 29944971 10.1016/j.humpath.2018.06.017

[4] Basu Mallick A, Chawla SP. Giant cell tumor of bone: an update. Curr Oncol Rep. 2021;23:51. 33754215 10.1007/s11912-021-01047-5

[5] Al-Ibraheemi A, Inwards CY, Zreik RT, Wenger DE, Jenkins SM, Carter JM, *et al*. Histologic spectrum of giant cell tumor (GCT) of bone in patients 18 years of age and below: a study of 63 patients. Am J Surg Pathol. 2016;40:1702–12. 27526293 10.1097/PAS.0000000000000715

[6] Roy S, Joshi NP, Sigamani E, Malik A, Sharma MC, Mohanti BK, *et al*. Clival giant cell tumor presenting with isolated trigeminal nerve involvement. Eur Arch Otorhinolaryngol. 2013;270:1167–71. 23143505 10.1007/s00405-012-2249-3

[7] Broehm CJ, Inwards CY, Al-Ibraheemi A, Wenger DE, Jenkins SM, Jin L, *et al*. Giant cell tumor of bone in patients 55 years and older: a study of 34 patients. Am J Clin Pathol. 2018;149:222–33. 29425276 10.1093/ajcp/aqx155

[8] Bihani A, Thiagarajan S, Chaukar D, D’Cruz AK. Giant cell tumor of hyoid bone: diagnostic dilemma with a novel management. J Cancer Res Ther. 2022;18:282–5. 35381802 10.4103/jcrt.JCRT_205_19

[9] Bahbah S, Harti KE, Wady WE. Giant cell tumor of the maxilla: an unusual neoplasm. Pan Afr Med J. 2020;36:342. 33224408 10.11604/pamj.2020.36.342.21919 PMC7664142

[10] Giri GV, Sukumaran G, Ravindran C, Narasimman M. Giant cell tumor of the mandible. J Oral Maxillofac Pathol. 2015;19:108. 26097323 10.4103/0973-029X.157217 PMC4451653

[11] Behjati S, Tarpey PS, Presneau N, Scheipl S, Pillay N, Van Loo P, Flanagan AM, *et al*. Distinct H3F3A and H3F3B driver mutations define chondroblastoma and giant cell tumor of bone. Nat Genet. 2013;45:1479–82. 24162739 10.1038/ng.2814 PMC3839851

[12] Kamble A, Hui M, Rao KN, Narayanan R, Reddy BR, Uppin SG, *et al*. Anti-histone H3.3 G34W antibody is a sensitive and highly specific immunohistochemistry marker for the diagnosis of Giant cell tumor of bone. A validation based on analysis of 198 cases from a single centre in India. Indian J Pathol Microbiol. 2022;65:617–29. 35900490 10.4103/ijpm.ijpm_265_21

[13] Schaefer IM, Fletcher JA, Nielsen GP, Shih AR, Ferrone ML, Hornick JL, *et al*. Immunohistochemistry for histone H3G34W and H3K36M is highly specific for giant cell tumor of bone and chondroblastoma, respectively, in FNA and core needle biopsy. Cancer Cytopathol. 2018;126:552–66. 29757500 10.1002/cncy.22000 PMC6441393

[14] Amary F, Berisha F, Ye H, Gupta M, Gutteridge A, Baumhoer D, *et al*. H3F3A (histone 3.3) G34W immunohaaistochemistry: a reliable marker defining benign and malignant giant cell tumor of bone. Am J Surg Pathol. 2017;41:1059–68. 28505000 10.1097/PAS.0000000000000859 PMC5510691

[15] Gomes CC, Diniz MG, Amaral FR, Antonini Guimarães BV, Gomez RS. The highly prevalent H3F3A mutation in giant cell tumours of bone is not shared by sporadic central giant cell lesion of the jaws. Oral Surg Oral Med Oral Pathol Oral Radiol. 2014;118:583–5. 25442495 10.1016/j.oooo.2014.07.011

[16] Hartmann W, Harder D, Baumhoer D. Giant cell-rich tumors of bone. Surg Pathol Clin. 2021;14:695–706. 34742488 10.1016/j.path.2021.06.010

[17] Nagar SR, Bansal S, Jashnani K, Sinha A, Desai RS. A comparative analysis of p63 expression in giant cell tumour (GCT), central giant cell granuloma (CGCG) and peripheral giant cell granuloma (PGCG). Head Neck Pathol. 2020;14:733–41. 31873936 10.1007/s12105-019-01118-x PMC7413967

[18] Miguita L, de Souza JC, Bastos VC, Pereira NB, de Freitas RAB, Guimarães LM, *et al*. Central giant cell granulomas of the jaws stromal cells harbour mutations and have osteogenic differentiation capacity, in vivo and in vitro. J Oral Pathol Med. 2022;51:206–16. 35048460 10.1111/jop.13274

[19] Baumhoer D, Amary F, Flanagan AM. An update of molecular pathology of bone tumors. lessons learned from investigating samples by next generation sequencing. Genes Chromosomes Cancer. 2019;58:88–99. 30582658 10.1002/gcc.22699

[20] Oliveira AM, Perez-Atayde AR, Inwards CY, Medeiros F, Derr V, Hsi BL, *et al*. USP6 and CDH11 oncogenes identify the neoplastic cell in primary aneurysmal bone cysts and are absent in so-called secondary aneurysmal bone cysts. Am J Pathol. 2004;165:1773–80. 15509545 10.1016/S0002-9440(10)63432-3 PMC3278819

[21] Klenke FM, Wenger DE, Inwards CY, Rose PS, Sim FH. Giant cell tumor of bone: risk factors for recurrence. Clin Orthop Relat Res. 2011;469:591–9. 20706812 10.1007/s11999-010-1501-7 PMC3018195

[22] Hayashida K, Kawabata Y, Kato I, Kamiishi T, Matsuo K, Takeyama M, *et al*. Clinical and pathological analysis of giant cell tumor of bone with denosumab treatment and local recurrence. J Orthop Sci. 2022;27:215–21. 33358447 10.1016/j.jos.2020.11.005

[23] Tariq MU, Umer M, Khan Z, Saeed J, Siddiqui MA, Din NU. Spectrum of histological features of denosumab treated giant cell tumor of bone; potential pitfalls and diagnostic challenges for pathologists. Ann Diagn Pathol. 2020;45:151479. 32088577 10.1016/j.anndiagpath.2020.151479

[24] Gilani A, Kleinschmidt-DeMasters BK. Denosumab therapy obscures histological features of giant cell tumor of bone. J Neuropathol Exp Neurol. 2019;78:1171–3. 31665371 10.1093/jnen/nlz100

